# B cells were related to HBsAg seroconversion in inactive HBsAg carriers following peginterferon therapy

**DOI:** 10.1371/journal.pone.0242559

**Published:** 2020-12-02

**Authors:** Zhenhuan Cao, Sha Meng, Yanhong Zheng, Junli Wang, Rui Wang, Xinyue Chen

**Affiliations:** 1 Fist Department of Liver Disease Center, Beijing You’an Hospital, Capital Medical University, Beijing, China; 2 Science and Technology Department, Beijing You’an Hospital, Capital Medical University, Beijing, China; 3 Beijing Key Laboratory for HIV/AIDS Research, Beijing, China; Centre de Recherche en Cancerologie de Lyon, FRANCE

## Abstract

Our recent study showed high rate of HBsAg seroconversion achieved in inactive HBsAg carriers (IHCs) treated with peginterferon (PEG-IFN). To better understand the immune-mediated component to the HBsAg seroconversion, we investigated the role of B cells in this study. A total of 44 IHCs were given 48 weeks of PEG-IFN. Fifteen cases achieve HBsAg seroconversion (R group), whereas 29 failed (NR group). The proportion of total B cells and plasma B cells were measured before and during treatment. We found that the proportion of total B cells and plasma B cells was no significant between R group and NR group at baseline, but significantly higher in R group than NR group during PEG-IFN treatment, even when the exact age-, sex-, and treatment period-match was made. In conclusion, we demonstrated the increase of total B cell and plasma B cells during PEG-IFN treatment favored HBsAg seroconversion for IHC, and B cells may play a role in HBV seroconversion.

## Introduction

Inactive HBsAg carriers (IHCs) characterized by effective control of HBV replication and sustained HBeAg seroconversion. Patients in this phase have detectable circulating HBsAg, absence of HBeAg, and presence of anti-HBe, as well as undetectable or low levels of HBV DNA. Our recent study showed high rate of HBsAg seroconversion for IHCs treated with peginterferon (PEG-IFN) [1]. Li et al also reported the similar finding in a small retrospective cohort study [2]. We also found a significant increase of B cells in patients who achieved HBsAg seroconversion.

The immune response of B cells is undoubtedly an important part of the body's protection against viral and bacterial infections by neutralizing, conditioning, antibody dependent cytotoxic effects and so on [3–5]. However, most reports focus on the relationship between HBV infection with T cell or NK cell at present [6, 7] and little attention paid to B cells, especially with HBsAg seroconversion. We therefore explore the contribution of B cells to HBsAg seroconversion in inactive HBsAg carriers following PEG-IFN.

## Materials and methods

### Patients

We enrolled IHC patients at Beijing You’an Hospital from Jan 1 to April 30, 2018. Peginterferon α-2a was administered 135μg/week for 48 weeks. The final date of follow up was July 16, 2019. At baseline and every 12 weeks thereafter during the treatment, detection of HBsAg levels, liver function, routine blood tests, and HBV DNA was performed, and peripheral blood mononuclear cells (PBMCs) were separated and stored at -80°C. We had access to information that could identify individual participants during and after data collection.

Inclusion criteria for IHC was HBsAg positive for more than 6 months, serum HBsAg levels <1000 IU/mL, HBeAg-negative/anti-HBe-positive, anti-HBc positive, HBV DNA<20IU/mL, absence of previous antiviral therapy within 1 year.

Exclusion criteria: human immunodeficiency virus or hepatitis C virus coinfections; history of autoimmune diseases; hepatic dysfunction; serious metabolic disease; cardiac or pulmonary or renal disease; evidence of liver carcinoma or other neoplastic diseases; contraindication for interferon.

Responder group (R): achieving HBsAg seroconversion. Non-responder group (NR): failed to achieve HBsAg seroconversion. Healthy control group (HC): HBsAg negative, vaccinated against HBV, and anti-HBs level >200 IU/L.

### Tests of HBV infection

HBsAg and anti-HBs levels were detected using the HBsAg quantitative Elecsys (Roche Diagnostics GmbH, German). The lower limit of quantitative HBsAg determination was 0.05 IU/mL, and anti-HBs >10 IU/L was defined as anti-HBs positive. HBV DNA levels were determined using the fluorescence quantitative polymerase chain reaction (FQ-PCR) method with a lower detection limit of 20IU/mL (Roche).

### Flow cytometry

**Fig 1:**
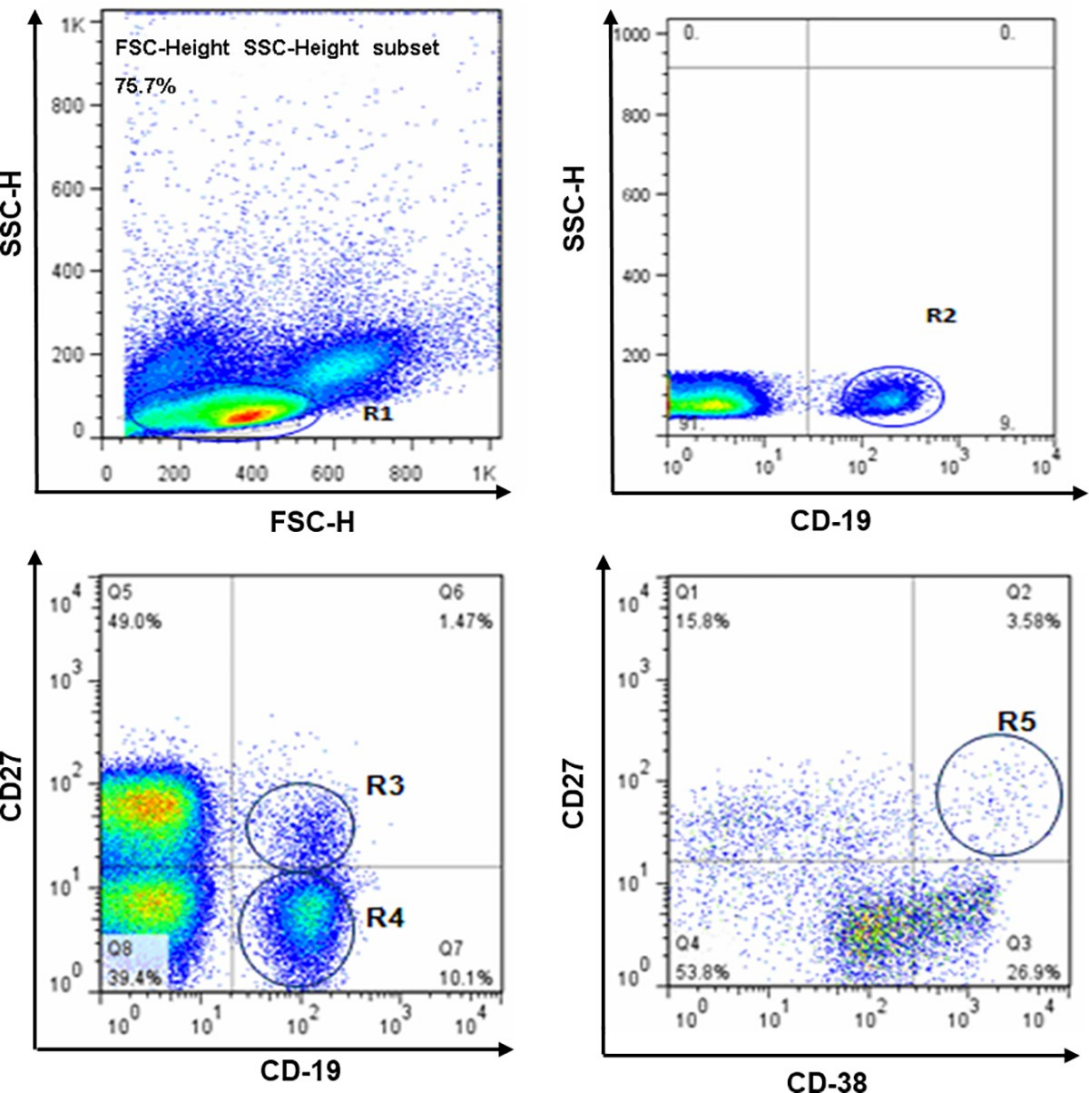
Analysis of B cells and plasma B cells. B cell and B cell subset PBMCs stained with CD19–APC, CD27-FITC, and CD38-PE gated on lymphocyte (R1), total B cells (R2, CD19+), naïve B cells (R3, CD19+CD27-), memory B cells (R4, CD19+CD27+), and then plasma B cells (R5, CD19+CD27hiCD38hi).

Monoclonal antibodies were added to the separated PBMCs in this order: CD19–APC, CD27-FITC and CD38-PE/CD80-PE. Isotype controls were also set up. FACS Calibur four-color flow cytometry was performed, and the results were analyzed using CELLQuest software (Becton Dickinson, San Jose, CA). All monoclonal antibodies were purchased from BD (San Diego, CA, USA). B cells were divided into four subsets: total B cells (CD19^+^), naïve B cells (CD19^+^CD27^-^), memory B cells (CD19^+^CD27^+^), and plasma B cells (CD19^+^CD27^hi^CD38^hi^). (Fig 1).

### Statistical analysis

Statistical analyses were performed using SPSS Version 24. All continuous data were present as mean ± standard deviation (SD). Categorical data were presented as number and percentages. Fisher’s exact or chi-square tests and t-test were conducted for the categorical and continuous variables, respectively, to compare the difference between variables and HBsAg seroconversion. The significance level was set at P<0.05 for all statistical analyses.

### Ethics approval

This study protocol was approved by the institutional ethics board of Beijing You’an Hospital, Capital Medical University (No. JYKLZ 2017 [24]).

## Results

### Patients’ clinical characterization

A total of 44 IHC patients were given 48 weeks of PEG-IFN. Fifteen cases achieve HBsAg seroconversion (R), whereas 29 failed (NR). There were no significant differences in sex, age, ALT, WBC and HBV DNA among R, NR and HC groups. The HBsAg level in R group was lower than that in NR group (p<0.001). Patients in R group experienced different periods of time to get HBsAg seroconversion. One case got it at 12 week after PEG-IFN therapy, and 7 cases, 2 cases, 5 cases obtained it at 24 week, 36 week and 48 week. The patients’ demographic and clinical data are listed in Table 1.

**Table 1:** Patients’ demographic and clinical data.

Parameter	R	NR	HC
	N = 15	N = 29	N = 12
gender (M/F)	8/7	17/12	6/6
Mean age, years ± SD	34.47±9.65	34.03 ±10.03	30.75±9.50
Mean ALT, units/L ± SD	27.25±9.26	25.87±8.80	30.54±6.19
Mean AST, units/L ± SD	25.53±5.44	23.49±6.34	20.58±6.31
Mean leukocyte,10^9^/L± SD	5.72±1.90	5.67±1.78	5.69±1.65
HBsAg, log10 IU/mL	1.33±0.96**	2.24±0.48**	UD
1^st^time achieved HBsAg seroconversion(n)			
12W	1	N	N
24W	7	N	N
36W	2	N	N
48W	5	N	N

** p<0.01; **R vs NR; UD = undetected.

### The proportion of total B cells and plasma B cells in IHCs and healthy control group at baseline

**Fig 2:**
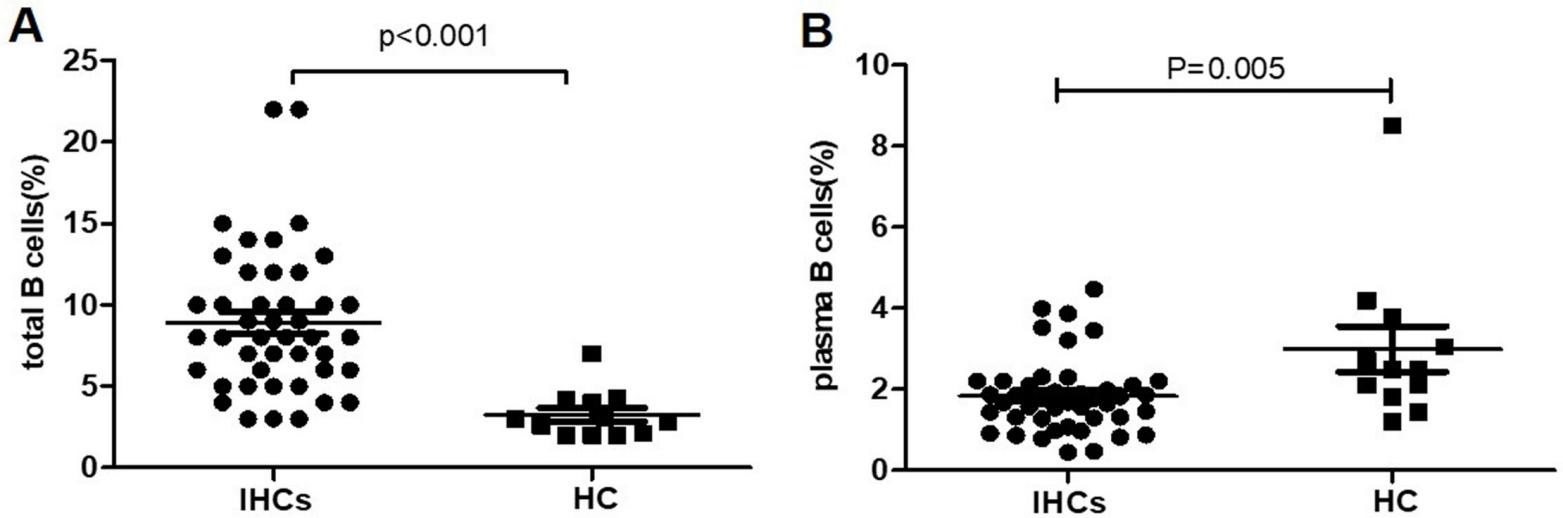
Comparison of total B and plasma B cells between IHCs and healthy control groups at baseline. (A) Comparison of total B cells subsets between IHCs (n = 44) and healthy controls (n = 12) at baseline. (B) Comparison of plasma B cells subsets between IHCs (n = 44) and healthy controls (n = 12) at baseline.

The proportion of total B cells (CD19^+^) and plasma B cells (CD19^+^CD27^hi^CD38^hi^) were analyzed by flow cytometry. The proportion of total B cells was (8.9±0.7)% in 44 IHCs at baseline, significantly higher than 12 healthy persons (3.2±0.4)%, *P*<0.001, Fig 2A. Differently, The proportion of plasma B cells was significantly lower in IHCs at baseline (1.8±0.1)% than healthy persons(3.0±0.6)%, *P* = 0.005. Fig 2B.

### The proportion of total B cells and plasma B cells in R and NR group at baseline

**Fig 3:**
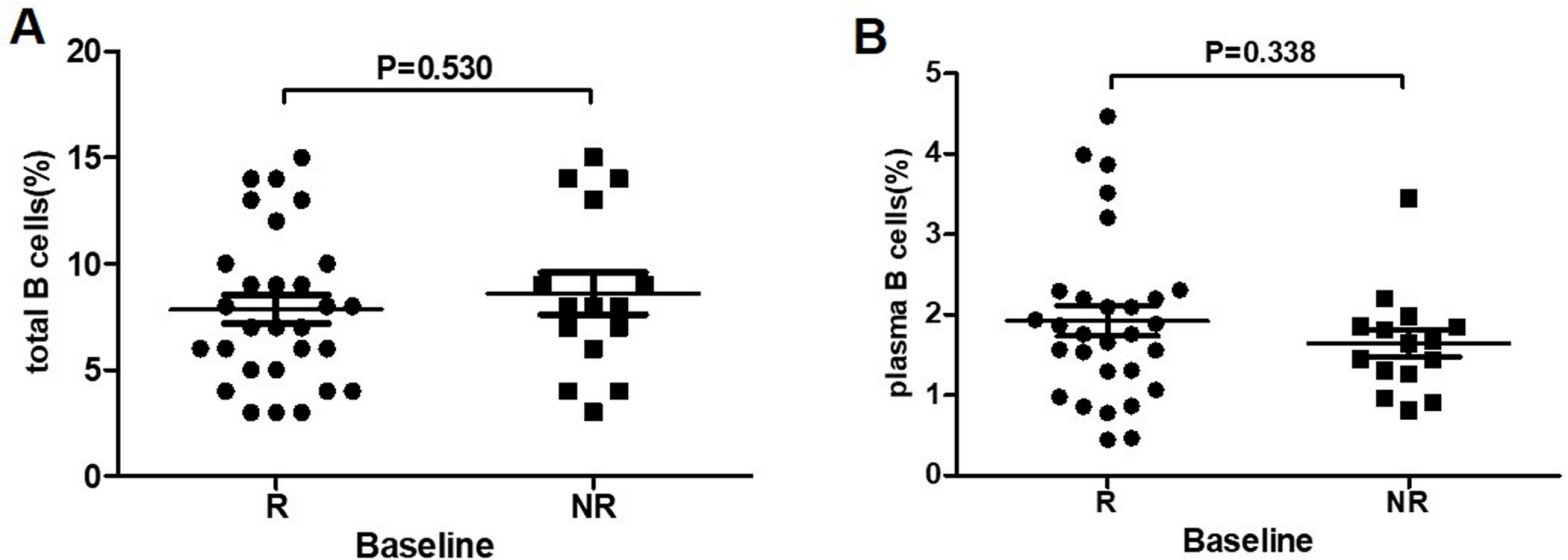
Comparison of total B and plasma B cells between R group and NR group at baseline. (A) Comparison of total B cells between IHCs who achieved HBsAg seroconversion (R group, n = 15) and who failed to achieve HBsAg seroconversion (NR group, n = 29) at baseline. (B) Comparison of plasma B cells between IHCs who achieved HBsAg seroconversion (R group, n = 15) and who failed to achieve HBsAg seroconversion (NR group, n = 29) at baseline.

After PEG-IFN treatment in 44 IHCs, 15 cases achieved HBsAg seroconversion (R group) and 29 unachieved (NR group). The proportion of total B cells was (7.9±0.7)% in R group, and ((8.6±1.0)% in NR group at baseline. There was no significant difference. Fig 3A. Similarly the proportion of plasma B cells was no significant difference between R group (1.9±0.2%) and NR group (1.6±0.2%), *P* = 0.338. Fig 3

### The proportion of total B cells and plasma B cells in age-, sex-, and treatment period-matched patients during PEG-IFN treatment in R and NR group

Because patients in R group (n = 15) experienced different periods of time to get HBsAg seroconversion, the comparison time points were set as the last time point before HBsAg seroconversion was detected (Pre-seroconversion point (Pre-P)) and the first time point when seroconversion of HBsAg was detected (Post-HBsAg seroconversion point (Post-P)). Given the fact that age and gender significantly affect the distribution of NK cells, 15 patients in NR group were compared with the 15 patients in R group after matching the age, sex, and course of treatment.

**Fig 4:**
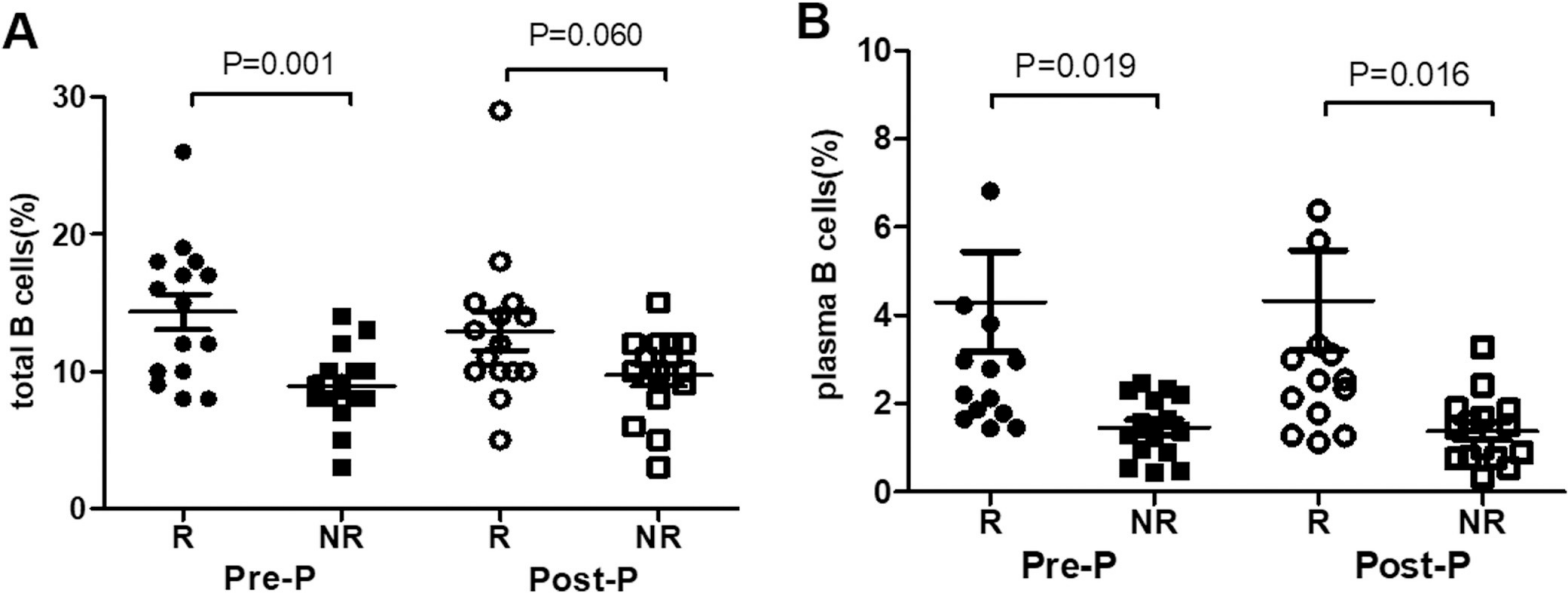
Comparison of total B and plasma B cells between R group and NR group during PEG-IFN treatment (A) Comparison of total B cells between IHCs who achieved HBsAg seroconversion (R group, n = 15) and who failed after matching the age, sex, and course of treatment (NR group, n = 15) at the time point before HBsAg seroconversion was detected (Pre-P) and at the time point when HBsAg seroconversion was detected (Post-P). (B) Comparison of plasma B cells between IHCs who achieved HBsAg seroconversion (R group, n = 15) and who failed after matching the age, sex, and course of treatment (NR group, n = 15) at Pre-SC, and Post-SC.

The results showed that the proportion of total B cells in R group was significantly higher than NR group at Pre-P (14.3±1.3 vs 8.9±0.7, *P* = 0.001), but no difference at Post-P (12.9±1.4 vs 9.7±0.8, *P* = 0.060), Fig 4. The proportion of plasma B cells were significantly higher in R group than NR group at Pre-P and Post-P (4.3±1.1 vs 1.5±0.2, *P* = 0.019; 4.3±1.1vs 1.4±0.2, *P* = 0.016), Fig 4B.

### The proportion of total B cells and plasma B cells in age-, sex-matched 7 patients achieved HBsAg seroconversion (R group) and 7 patients failed (NR group) at 24 week of PEG-IFN treatment

Considering the differences in clinical characterization and length of time needed to achieve HBsAg seroconversion in the R group, we further selected seven patients who achieved HBsAg seroconversion at 24 weeks, and seven age/sex/HBsAg level at baseline -matched NR cases with for comparison (Table 2).

**Table 2:** Clinical characterization of seven patients who achieved HBsAg seroconversion at 24 weeks, and seven age/sex/HBsAg level at baseline -matched NR cases.

	Patient ID	Age	Sex	ALT	HBsAg	1^st^time point achieved seroconversion
R (n = 7)	1	32	M	32	239.3	24W
	2	39	M	29	0.693	24W
	3	39	M	35	2.85	24W
	4	17	F	33	0.326	24W
	5	40	F	31	13.56	24W
	6	32	F	30	85	24W
	7	41	M	28	70	24W
NR (n = 7)	1	38	M	28	230.4	NA
	2	35	M	29	1.424	NA
	3	40	M	31	6.7	NA
	4	20	F	34	0.592	NA
	5	36	F	29	12.9	NA
	6	32	M	30	89.4	NA
	7	40	F	34	78	NA

NA = not achived.

**Fig 5:**
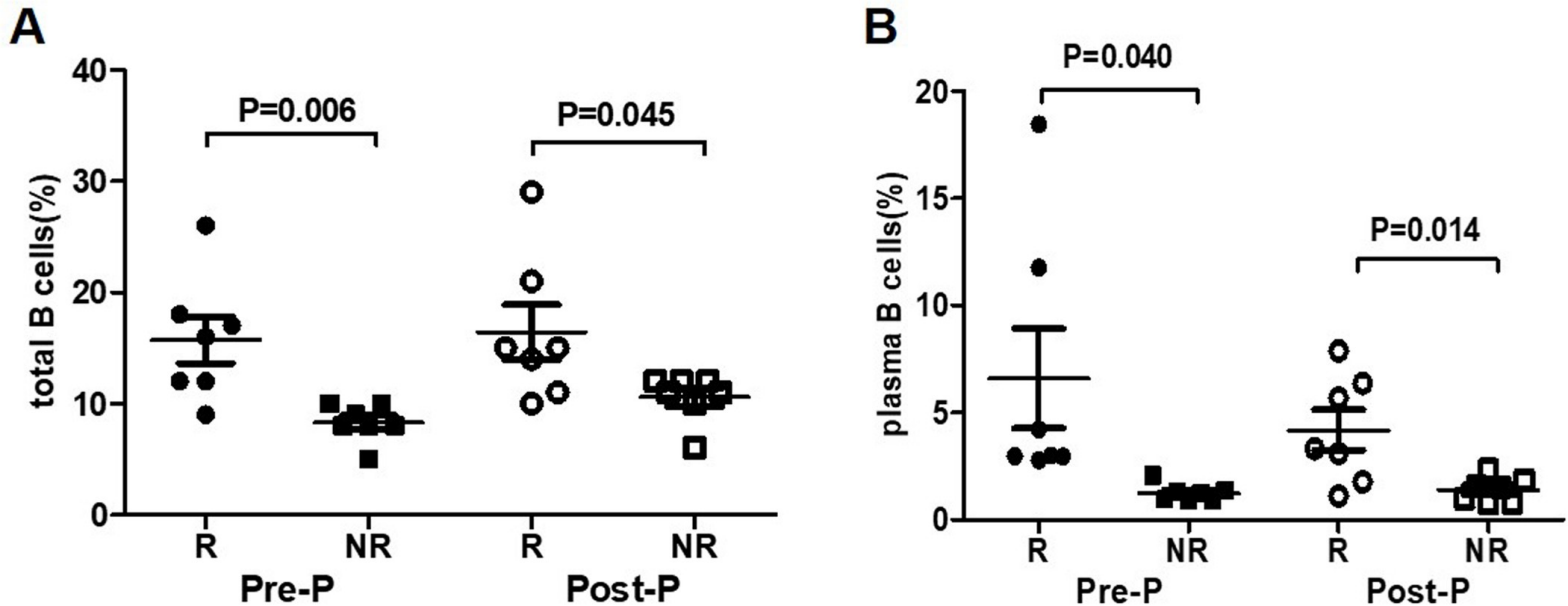
Comparison of total B and plasma B cells between R group and NR group during PEG-IFN treatment (A) Total B cells in age-, sex-, and HBsAg level at baseline -matched patients: comparison between IHCs who achieved seroconversion at 24 weeks of treatment (n = 7) and the patients who failed at 24 weeks of treatment (n = 7). (B) Plasma B cells in age-, sex-, and HBsAg level at baseline-matched patients: Comparison between IHCs who achieved HBsAg seroconversion at 24 weeks of treatment (n = 7) and the patients who failed at 24 weeks of treatment (n = 7).

The results showed the proportion of total B cells in R group were significantly higher than NR group at Pre-P (12 weeks of PEG-IFN treatment) and Post-P (24 weeks of PEG-IFN treatment), (15.7±2.1 vs 8.3±0.6, *P* = 0.006; 16.4±2.5 vs 10.6±0.8, *P* = 0.045) Fig 5A. Similarly, the proportion of plasma B cells in R group were significantly higher than NR group at Pre-P and Post-P (6.6±2.3 vs 1.2±0.2, *P* = 0.040; 4.2±0.9 vs 1.4±0.2, *P* = 0.014), Fig 5.

## Discussion

In our recent study, we observed a high proportion of HBsAg seroconversion cases in IHC patients treated with PEG-IFN [1]. The exact mechanism of how PEG-IFN promotes HBsAg clearance remains unknown. The predictive factors of HBsAg clearance are still being explored. In the process of eliminating HBV infection, adaptive immune response plays a crucial role. But in this process, T cell function is depleted [8, 9]. Antibodies to HBsAg are particularly important. B cells have been proved to play a crucial role in controlling HBV infection [10, 11]. However, the B cell mediated immune response in HBV clearance remains unclear. Therefore, it is very important to explore the relationship between B cell and HBV infection, especially HBsAg seroconversion. Here, we performed longitudinal analysis of lymphocyte subsets in IHC patients undergoing PEG-IFN treatment focusing on B cells.

HBV infection is usually associated with increased B cell activation and chemotaxis, and activated memory B cells display enhanced differentiation into immunoglobulin-producing cells. However, activated B cells display significantly lower proliferative ability [12], so the induced immune response does not last very long. Perhaps this is the cause of chronicity following HBV infection. The percentage of memory B lymphocytes significantly decreases in HBV-related liver cancer patients relative to healthy controls [13], suggesting a correlation of total B cells and the distribution of different B cells to disease outcome. In agreement with published CHC studies [14–16], we found that the proportion of total B cells at baseline was higher in the IHCs than in the healthy group.

In our study, the proportion of total and plasma B cells were similar with patients who achieve HBsAg seroconversion and not. Dynamic changes in total and plasma B cells were analyzed in IHC patients underwent PEG-IFN therapy, and we found a gradual increase in total B cells during treatment, particularly at the last time point before HBsAg seroconversion (Pre-P) compared to the NR group. Considering the differences in clinical characterization and length of time to achieve HBsAg seroconversion, we further selected seven patients who achieved HBsAg seroconversion at 24 weeks, and seven matched NR cases with for comparison. The results also showed the proportion of total B and plasma cells were significantly higher in HBsAg seroconversion patients than NR group at Pre-P and at the first time point when seroconversion of HBsAg was detected (Post-P).

Consistent with our study, Xu X et al comprehensively investigated the frequency, phenotype, and function of peripheral B-cell subsets from chronic hepatitis B patients in different phases. They found the frequency of HBsAg-specific B cells were significantly decreased in CHB patients compared with healthy controls. More importantly, they reported IFN-α might preferentially activate B cells, and the reversal of B-cell functional impairment was associated with HBsAg seroconversion [17]. Reconstitution of anti-HBs response represents a rational therapy for chronic HBV infection.

Taken together, we found that the increases in total B cell and plasma B cells during the course of PEG-IFN treatment. The peaks of both total B cell and plasma cell levels coincided with HBsAg seroconversion, strongly suggesting the important role of B cells in HBV virus clearance. Our study highlights the therapeutic role of neutralizing anti-HBs against chronic HBV infection.

## Supporting information

S1 Data (XLSX)
